# Integrative multi-omics reveals genetic and transcriptomic determinants of aroma formation during alcoholic fermentation in *Saccharomyces cerevisiae*

**DOI:** 10.3389/fmicb.2026.1866172

**Published:** 2026-05-29

**Authors:** Inseo Kim, Sung Han Kim, Soo Kweon Lee, Eunkyung Choi, Soyun Choi, Doyoon Shin, Ahyoung Lim, Seokmin Yoon, Hyun Park

**Affiliations:** 1 Department of Biotechnology, College of Life Sciences and Biotechnology, Korea University, Seoul, Republic of Korea; 2 LOTTE R&D Center, Seoul, Republic of Korea; 3 Department of Bioprocess Technology, Bio Campus of Korea Polytechnics, Nonsan, Republic of Korea

**Keywords:** aroma metabolism, multi-omics analysis, *Saccharomyces cerevisiae*, volatile organic compounds, yeast aroma biosynthesis

## Abstract

Aroma-active volatile organic compounds (VOCs) produced during alcoholic fermentation by *Saccharomyces cerevisiae* are key determinants of the sensory quality of fermented beverages. However, the molecular mechanisms underlying strain-specific aroma diversity remain incompletely understood. Thus, this study aimed to integrate genomic, transcriptomic, and metabolomic analyses to elucidate the genetic and regulatory determinants of VOC biosynthesis in three *S. cerevisiae* strains. Whole-genome sequencing identified genomic variation among strains, whereas RNA sequencing (RNA-seq) analyses revealed distinct transcriptional profiles among the strains. Gas chromatography–mass spectrometry (GC–MS) profiling identified strain-dependent differences in VOC composition, including higher alcohols, esters, and acids. Integrative analysis demonstrated that elevated expression of *ADH1*, *ADH3*, and *ADH5* in strain SC8292 was associated with production of higher alcohols via the Ehrlich pathway, whereas upregulation of *EEB1* and *EHT1* promoted fatty acid ester synthesis. In contrast, strain SC8293 showed strong expression of *ADH2* and *PDC1*, which coincided with acetaldehyde accumulation, whereas strain SC8301 was enriched in acetate esters, contributing to fruity aromas. Gene synteny analysis revealed the absence of *ALD2* in SC8292, explaining the lack of acetic acid production in this strain. Furthermore, transcription factors including GCR1, ADR1, and SIP4 were implicated in regulating VOC biosynthesis. Collectively, these findings demonstrate that genomic variation and transcriptional regulation jointly shape strain-specific aroma profiles. The integration of multi-omics datasets provides mechanistic insight into the molecular basis of aroma diversity and identifies candidate targets for strain selection and metabolic engineering to improve fermentation flavor quality.

## 1 Introduction

Fermentation by *Saccharomyces cerevisiae* is one of the most important biotechnological processes, responsible for producing alcoholic beverages, bread, and biofuels. In particular, during alcoholic fermentation, yeast converts sugars into ethanol and generates a wide range of secondary metabolites, among which volatile organic compounds (VOCs) are the primary contributors to the aroma and flavor characteristics of fermented products. VOCs include higher alcohols, esters, acids, and aldehydes, whose concentrations and relative ratios strongly influence sensory perception. For example, acetate esters such as isoamyl acetate and phenethyl acetate impart fruity and floral aromas (Vilela, 2020). In contrast, excessive levels of fatty acids or acetic acid are associated with off-flavors described as rancid, sour, or sweaty (Tekarslan-Sahin, 2022).

The biosynthetic routes underlying VOC production are linked to central carbon and amino acid metabolism. The Ehrlich pathway converts amino acids into higher alcohols via sequential transamination, decarboxylation, and reduction steps, and has been studied extensively in yeast (Hazelwood et al., 2008). Esters are formed through the action of alcohol acetyltransferases (ATF1 and ATF2) (Verstrepen et al., 2003) and fatty acyl transferases encoded by the *EEB1* gene, encoding ethyl ester biosynthesis protein 1, and the *EHT1* gene, encoding ethanol hexanoyl transferase 1, (Saerens et al., 2006), which condense alcohols with acyl-CoA derivatives. Acetic acid is produced from pyruvate via aldehyde dehydrogenases (*ALD2* and *ALD6*) (Eglinton et al., 2002). These pathways are subject to both genetic variation among strains and transcription factors such as RAP1, GCR1, ADR1, and SIP4 (Santangelo, 2006). Although the biochemical pathways involved in VOCs biosynthesis have been extensively characterized, substantial strain-dependent variation in volatile metabolite production remains poorly understood. Different *S. cerevisiae* strains can produce markedly distinct aroma profiles even under identical fermentation conditions, reflecting underlying genetic and regulatory diversity (Mendes et al., 2017). Large-scale genomic studies have revealed extensive genetic variation among domesticated yeast strains used in industrial fermentation processes, suggesting that differences in gene content, regulatory networks, and enzyme activity may contribute to variation in metabolite production (Peter et al., 2018). Thus, elucidating how genetic diversity translates into metabolic phenotypes remains an important challenge in fermentation biology.

Recent advances in high-throughput sequencing and analytical technologies have enabled the application of multi-omics approaches to investigate microbial metabolism at the systems level. Transcriptomic analyses provide insights into gene expression patterns underlying metabolic processes, while metabolomic profiling enables direct measurements of biochemical outputs such as volatile compounds. However, many previous studies have examined these datasets independently, limiting the ability to establish mechanistic links between gene expression and metabolite production (Tzamourani et al., 2025). Integrating genomic, transcriptomic, and metabolomic datasets offers a powerful framework for identifying gene–metabolite relationships and for uncovering regulatory mechanisms that control aroma biosynthesis.

The present study applied an integrative multi-omics approach that combined whole-genome sequencing, transcriptome analysis, and GC–MS based volatile metabolite profiling to investigate aroma formation in three industrial *S. cerevisiae* strains used for alcoholic fermentation. By comparing genomic variation, transcriptional patterns, and volatile metabolite production among these strains, we aimed to identify key genes and metabolic pathways underlying strain-specific aroma phenotypes. This integrative analysis provides new insights into the molecular determinants of aroma metabolism in yeast and highlights potential molecular targets for developing industrial strains with enhanced flavor-producing capabilities.

## 2 Materials and methods

### 2.1 Yeast strains and growth conditions

*Saccharomyces cerevisiae* strains 8292 (SC8292), 8293 (SC8293), and 8301 (SC8301), isolated from nuruk, a traditional Korean fermentation starter, were provided by the LOTTE R&D Center (Seoul, Republic of Korea) for use in this study. These strains were selected by the LOTTE R&D Center based on the associated superior ethanol production performance in distilled liquor fermentations and are currently the subject of a patent application. The yeast strains were stored at −80 °C until use, then activated prior to fermentation by cultivation on yeast extract–peptone–dextrose (YPD) agar (1% yeast extract, 2% peptone, 2% glucose) and incubation at 30 °C for 24 h. A single colony from each plate was inoculated into 50 mL of YPD broth and cultivated at 30 °C for 24 h; the resulting cultures were used as seed inocula for fermentation experiments.

### 2.2 Whole genome sequencing, assembly and annotation

Genomic DNA was isolated using a standard phenol–chloroform extraction protocol (Dymond, 2013). Quantitative and qualitative assessment of the extracted DNA was performed using a Qubit 2.0 Fluorometer (Invitrogen, Thermo Fisher Scientific, Waltham, MA, United States) to confirm appropriate nucleic acid concentration. Genomic DNA that passed quality control criteria was sheared to an average fragment size using a Megaruptor v3 (Diagenode, Liège, Belgium). The sheared DNA was purified with 1 × SMRTbell cleanup beads, and the fragment size distribution was verified using a Femto Pulse system (Agilent Technologies, Santa Clara, CA, United States). DNA repair and A-tailing were performed by incubating the sheared DNA with Repair Buffer, End Repair Mix, and DNA Repair Mix. Subsequently, SMRTbell Hairpin Adapters were ligated to the repaired DNA fragments using Ligation Mix and Ligation Enhancer at 20 °C for 30 min. The resulting library was purified with 1 × SMRTbell cleanup beads. Genomes of *S. cerevisiae* strains were sequenced on the PacBio RS II platform (Pacific Biosciences, Menlo Park, CA, United States). The approximate genome coverage depths for SC8292, SC8293, and SC8301 were 360×, 54×, and 349×, respectively. *De novo* genome assembly was performed using PacBio long-read sequencing data with hifiasm (v0.16.1-r375). Assembly completeness was assessed with Benchmarking Universal Single-Copy Orthologs [MOU16.1] (BUSCO; v5.6.1) using the Ascomycota_odb10 dataset (Manni et al., 2021). All gene prediction data were integrated using EVidenceModeler (v2.1.0, EVM), which is widely used for eukaryotic gene prediction (Haas et al., 2008). Functional annotation was conducted using BlastP (v2.2.29) based on the *S. cerevisiae* RefSeq database from the National Center for Biotechnology Information (NCBI) (Altschul et al., 1990). Gene Ontology (GO) analysis was performed using OmicsBox software (v2.1.2) (Conesa et al., 2005).

### 2.3 Yeast fermentation and volatile compounds analysis

Yeast strains were pre-cultured in YPD medium at 30 °C for 24 h, harvested by centrifugation (8,000 × g, 10 min), washed twice with sterile 0.85% saline, and resuspended to 2.0 × 10^8^ cells/mL based on counts obtained using a hemocytometer (Marienfeld Superior, Germany). A rice saccharified solution was prepared using rice koji as the saccharifying agent, following a previously reported method. Briefly, rice koji (Hwangguk rice koji; Suwon Fermentation Inc., Korea) and α-gelatinized rice flour (Alphami powder, Milplus AM01; Hyunjin Greenmill Co., Ltd., Korea) were mixed with distilled water at a ratio of 3:7:21 (w/w/w) and allowed to hydrate for 1 h. Saccharification was then performed at 56 °C for 8 h, and the saccharified solution was adjusted to 10° Brix before fermentation. The yeast suspension was inoculated into the rice saccharified solution at 0.5% (v/v), resulting in an initial cell density of 1.0 × 10^6^ cells/mL. Fermentation was conducted under static conditions at 30 °C for 48 h. Following fermentation, cultures were centrifuged (8,000 × g, 10 min), and the supernatants were collected after filtration through Whatman No. 2 filter paper (Whatman No. 2, Cytiva, Marlborough, MA, United States). All fermentation experiments were conducted in triplicate (*n* = 3) using 250 mL bottles (DURAN® Original GL45, DWKLife Sciences GmbH, Wertheim, Germany) containing 200 mL of the medium.

Volatile compounds in fermented rice saccharified solutions were analyzed by solid-phase microextraction coupled with gas chromatography–mass spectrometry (SPME–GC–MS) according to previously reported methods with minor modifications (Lee et al., 2016). Briefly, 3 g of each sample was transferred into a 20 mL headspace vial and equilibrated at 40 °C for 30 min. Volatile compounds in the headspace were extracted using an 85 μm CAR/PDMS fiber (Supelco, Bellefonte, PA, United States) for 30 min. GC–MS analysis was performed on an Agilent 7890B gas chromatograph coupled to a 5977A mass selective detector (Agilent Technologies, Santa Clara, CA, United States). Separation was achieved on a DB-WAX capillary column (30 m × 0.25 mm i.d., 0.25 μm film thickness, Agilent Technologies, Santa Clara, CA, United States) using helium as the carrier gas at a constant flow rate of 1.0 mL/min. The injector temperature was set to 250 °C, and the oven temperature was initially held at 40 °C for 2 min, increased at a rate of 2 °C/min to 220 °C, followed by a ramp of 20 °C/min to 240 °C, and maintained at 240 °C for 5 min. Mass spectra were acquired in electron ionization (EI) mode at 70 eV over an m/z range of 35–450. Volatile compounds were tentatively identified by comparing mass spectra with the Wiley 9NIST 0.8 spectral library (version 5.0) as described by Choi et al. (2024). Relative quantification was performed based on peak area normalization, and results are expressed as the percentage of the total peak area. Calibration curve-based absolute quantification was not performed in this study, as the GC–MS analysis was designed for exploratory comparative profiling of strain-specific VOC patterns. All analyses were performed in triplicate, and statistical significance was determined by one-way analysis of variance (ANOVA) with Tukey’s *post hoc* test (*p* < 0.05) using GraphPad Prism (version 9; GraphPad Software, Boston, MA, United States).

### 2.4 RNA sequencing and transcriptome analyses

Total RNA was extracted using hot acidic phenol (Collart and Oliviero, 1993). The quality and integrity of the total RNA were assessed using an Agilent Technologies 2100 Bioanalyzer (Agilent Technologies, Santa Clara, CA, United States). The purity of RNA samples was evaluated using a NanoDrop 8000 spectrophotometer (Thermo Fisher Scientific, Waltham, MA, United States). A transcriptome sequencing (RNA-seq) library was prepared using the TruSeq Stranded mRNA Library Prep kit (Illumina, San Diego, CA, United States). After enriching the mRNA with RNA purification beads, the mRNA was fragmented, and first-strand cDNA synthesis was performed using reverse transcriptase and random hexamer primers (Cui et al., 2023). The second strand of the cDNA was then synthesized, A-tailed, and ligated to sequencing adapters (Peng et al., 2023). Adapter-ligated products were then amplified by PCR, and the resulting cDNA libraries were sequenced on an Illumina NovaSeq 6000 platform (Illumina, San Diego, CA, United States). RNA-seq reads were trimmed to remove adapter sequences and aligned to the reference genome using TopHat (v2.1.1) (Trapnell et al., 2009). Differential gene expression analysis was performed using Cufflinks (v2.2.1) (Trapnell et al., 2012). To improve the accuracy of identifying differentially expressed genes (DEGs), genes with |log_2_FC| > 1 were defined as significant DEGs. RNA-seq analysis was performed to identify transcriptional changes between non-fermented control and fermented conditions for each strain. Because transcriptomic analysis was conducted using single biological samples for each condition, the RNA-seq results should be interpreted as an exploratory analysis intended to identify potential molecular associations.

GO analysis was performed to functionally categorize DEGs and identify enriched biological processes. GO annotations provided insights into the potential roles of DEGs, while enrichment analysis revealed significantly overrepresented functional categories. Additionally, Kyoto Encyclopedia of Genes and Genomes (KEGG) pathway analysis was conducted to interpret high-throughput molecular data and elucidate the systemic biological functions and interactions of DEGs. To identify correlated genes and metabolites and examine the associated relationships, two analytical models were applied based on gene expression and metabolite abundance data. First, a pathway-based functional model was used to identify shared KEGG metabolic pathways and assess correlation patterns between genes and metabolites. Second, an orthogonal projections to latent structures (O2PLS) model was constructed to integrate gene and metabolite data, enabling the joint analysis of correlated gene–metabolite pairs based on predictive components.

## 3 Results

### 3.1 General genomic characteristics

All three yeast genomes were sequenced using the PacBio sequencing technologies. The final assemblies ranged in size from 11.92 to 11.99 Mbp, with N50 contig lengths of 888–923 kb. The average GC contents were 38.22–38.27%. On average, 5,549 protein-coding genes were predicted in the genomes of strains SC8292, SC8293, and SC8301 (Table 1). Assembly completeness was confirmed using BUSCO; among the 1706 Ascomycota orthologous genes, 1,660 (97.3%) to 1,663 (97.5%) were identified as complete genes (Table 2).

**Table 1:** Statistics of sequencing, assembly, and annotation of genomes.

Strain		SC8292	SC8293	SC8301
Sequencing	Number of HiFi reads	497,970	85,682	501,320
	Average length of HiFi reads, nt	8,668	7,484	8,321
Assembly	Total size of contigs, bp	11,997,721	11,924,194	11,941,676
	Longest contig, bp	1,491,483	1,477,316	1,512,517
	N50 contig length, bp	923,785	888,502	896,958
	% GC contents	38.22	38.26	38.27
Annotation	Number of genes	5,535	5,565	5,548
	Total gene length, bp	8,607,512	8,620,365	8,639,193
	Average gene length, bp	1,555	1,549	1,556
	Number of CDS	5,535	5,565	5,548
	Total CDS length, bp	8,541,196	8,556,402	8,566,907
	Average CDS length, bp	1,543	1,538	1,544

**Table 2:** BUSCO analysis of assembled genomes.

Strain	SC8292	SC8293	SC8301
Complete BUSCOs	1,663 (97.5%)	1,660 (97.3%)	1,663 (97.5%)
Complete and single-copy BUSCOs	1,579 (92.6%)	1,534 (89.9%)	1,557 (91.3%)
Complete and duplicated BUSCOs	84 (4.9%)	126 (7.4%)	106 (6.2%)
Fragmented BUSCOs	5 (0.3%)	8 (0.5%)	7 (0.4%)
Missing BUSCOs	38 (2.2%)	38 (2.2%)	36 (2.1%)

### 3.2 Impact of fermentation on VOC production

**Figure 1:**
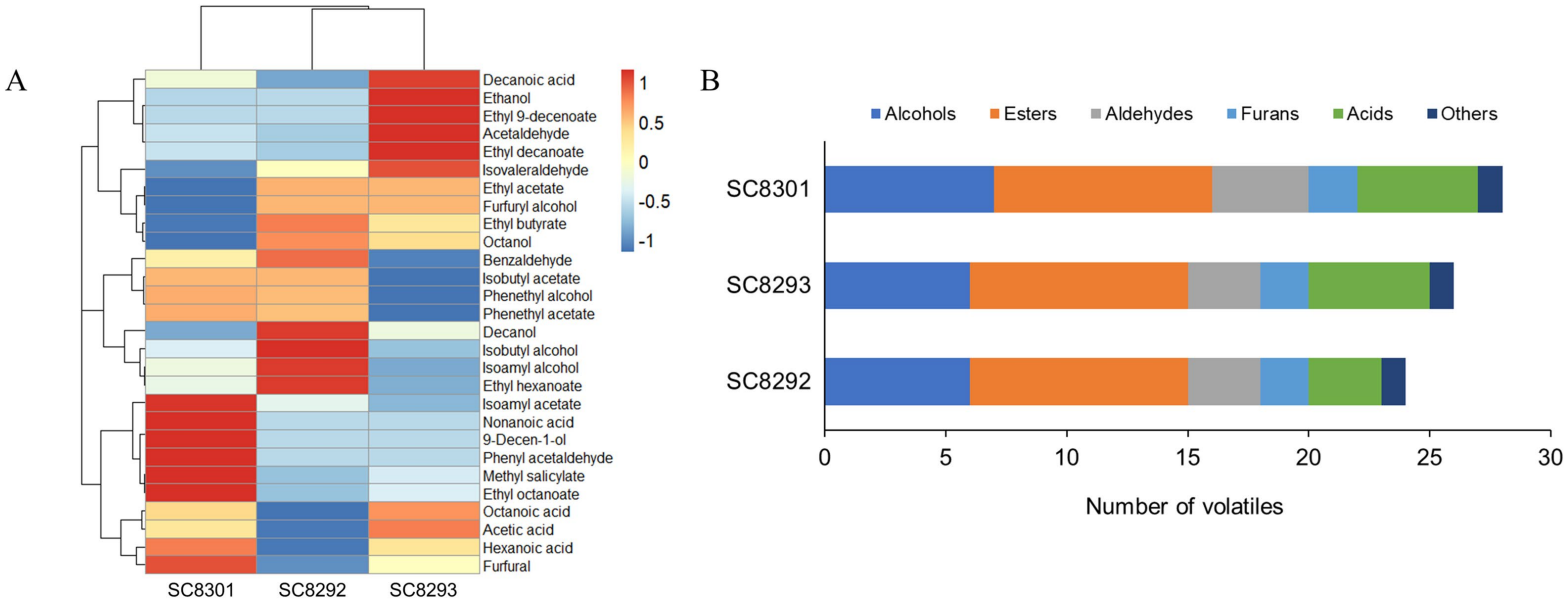
Comparative profiling of volatile organic compounds (VOCs) produced by three *Saccharomyces cerevisiae* strains during alcoholic fermentation. **(A)** Heatmap showing the relative abundance of VOCs detected in fermentation samples from three *S. cerevisiae* strains (SC8292, SC8293, and SC8301) analyzed by gas chromatography–mass spectrometry (GC–MS). Color intensity indicates the normalized abundance of each compound across strains. Distinct clustering patterns highlight strain-dependent differences in aroma metabolite production. **(B)** Classification of identified VOCs according to chemical categories. A total of 28 volatile compounds were detected and grouped into six major classes: acids, alcohols, aldehydes, esters, furans, and others.

To investigate strain-dependent differences in aroma formation during alcoholic fermentation, VOCs produced by the three *S. cerevisiae* strains were analyzed using GC–MS. A total of 28 volatile compounds were identified and classified into six categories: acids, alcohols, aldehydes, esters, furans, and others (Table 3). Among these compounds, esters and alcohols were the most prevalent, followed by acids, aldehydes, furans, and other classes (Figure 1B). Heatmap analysis revealed significant differences in VOC composition among *S. cerevisiae* strains (Figure 1A). Notably, acetate esters (isobutyl acetate, isoamyl acetate, and phenethyl acetate), which are key contributors to fruity aroma, were most abundant in SC8301, suggesting enhanced esterification activity under the associated fermentation conditions. In contrast, SC8292 produced relatively higher levels of higher alcohols, which contribute to fermented and floral notes and are derived from amino acid catabolism. SC8293 generated markedly higher levels of acid-derived volatiles, particularly acetic acid and hexanoic acid, than the other strains. These compounds are commonly associated with unpleasant off-flavors, such as sour, rancid, or sweaty aromas, especially when present at elevated concentrations (Table 3). Collectively, these differences highlight the impact of yeast strain selection on the final VOC profile of fermented products.

**Table 3:** Volatile profiles of the three *S. cerevisiae* strains.

Classification	Name	CAS No.	Relative peak area (%)*		
			SC8292	SC8293	SC8301
Acids	Hexanoic acid	142-62-1	0.78 ± 0.10^a^	0.95 ± 0.05^a^	1.01 ± 0.18^a^
	Octanoic acid	124-07-2	1.33 ± 0.23^a^	2.50 ± 0.03^b^	2.30 ± 0.08^b^
	Nonanoic acid	112-05-0	0.11 ± 0.00^a^	0.11 ± 0.01^a^	0.12 ± 0.04^a^
	Decanoic acid	334-48-5	ND	0.33 ± 0.03^b^	0.12 ± 0.05^a^
	Acetic acid	64-19-7	ND	0.68 ± 0.04^b^	0.49 ± 0.01^a^
Alcohols	Isobutyl alcohol	78-83-1	0.64 ± 0.30^a^	0.41 ± 0.34^a^	0.45 ± 0.01^a^
	Isoamyl alcohol	123-51-3	20.06 ± 0.16^b^	15.57 ± 0.04^a^	17.03 ± 0.03^a^
	9-decen-1-ol	13019-22-2	ND	ND	0.03 ± 0.01
	Phenethyl alcohol	60-12-08	9.94 ± 0.13^b^	3.51 ± 1.29^a^	10.27 ± 1.97^b^
	Octanol	111-87-5	0.28 ± 0.17^a^	0.26 ± 0.21^a^	0.18 ± 0.01^a^
	Ethanol	64-17-5	46.31 ± 0.21^a^	59.50 ± 1.01^b^	46.26 ± 1.38^a^
	Decanol	112-30-1	0.06 ± 0.01^a^	0.04 ± 0.01^a^	0.03 ± 0.02^a^
Aldehydes	Acetaldehyde	75-07-0	0.27 ± 0.02^a^	0.73 ± 0.10^b^	0.31 ± 0.02^a^
	Benzaldehyde	100-52-7	3.18 ± 1.09	1.04 ± 0.02	2.40 ± 0.04
	Phenyl acetaldehyde	122-78-1	ND	ND	0.04 ± 0.01
	Isovaleraldehyde	590-86-3	0.07 ± 0.00^b^	0.10 ± 0.01^c^	0.04 ± 0.01^a^
Esters	Isobutyl acetate	110-19-0	0.06 ± 0.00^a^	0.05 ± 0.01^a^	0.06 ± 0.01^a^
	Isoamyl acetate	123-92-2	2.67 ± 0.03	1.60 ± 0.14	5.88 ± 0.81
	Phenethyl acetate	103-45-7	0.30 ± 0.04^a^	0.12 ± 0.00^a^	0.31 ± 0.28^a^
	Ethyl acetate	141-78-6	3.40 ± 2.06^a^	3.39 ± 0.58^a^	2.79 ± 0.01^a^
	Ethyl butyrate	105-54-4	0.57 ± 0.04^a^	0.49 ± 0.02^a^	0.28 ± 0.12^a^
	Ethyl hexanoate	123-66-0	3.52 ± 0.13^b^	1.96 ± 0.16^a^	2.42 ± 0.01^a^
	Ethyl octanoate	106-32-1	1.33 ± 0.00^a^	1.36 ± 0.02^a^	1.50 ± 0.17^a^
	Ethyl decanoate	110-38-3	0.04 ± 0.00^a^	0.15 ± 0.01^c^	0.05 ± 0.01^b^
	Ethyl 9-decenoate	67233-91-4	0.04 ± 0.01^a^	0.07 ± 0.02^a^	0.04 ± 0.01^a^
Furans	Furfural	98-01-01	0.09 ± 0.01^a^	0.10 ± 0.01^a^	0.11 ± 0.01^a^
	Furfuryl alcohol	98-00-0	0.16 ± 0.00^a^	0.16 ± 0.00^a^	0.14 ± 0.02^a^
Others	Methyl salicylate	119-36-8	4.96 ± 2.66^a^	5.01 ± 0.31^a^	5.27 ± 1.97^a^

*Data are expressed as the mean ± standard error of the mean; ^abc^ indicate significant differences among groups in the same row (*p* < 0.05); ND, not detected.

### 3.3 DEG analysis

Comparative transcriptomic analyses were performed among the three strains to determine whether the observed differences in VOC production were associated with distinct transcriptional programs. To improve the accuracy of DEG identification, genes with |log_2_FC| ≥ 1 were defined as DEGs. A total of 1988, 1,416, and 1,184 DEGs were identified in the SC8292, SC8293, and SC8301 strains, respectively. In SC8292, 984 genes were upregulated, and 1,004 were downregulated. In SC8293, 774 genes were upregulated, and 642 were downregulated. In SC8301, 615 genes were upregulated, and 568 were downregulated.

**Figure 2:**
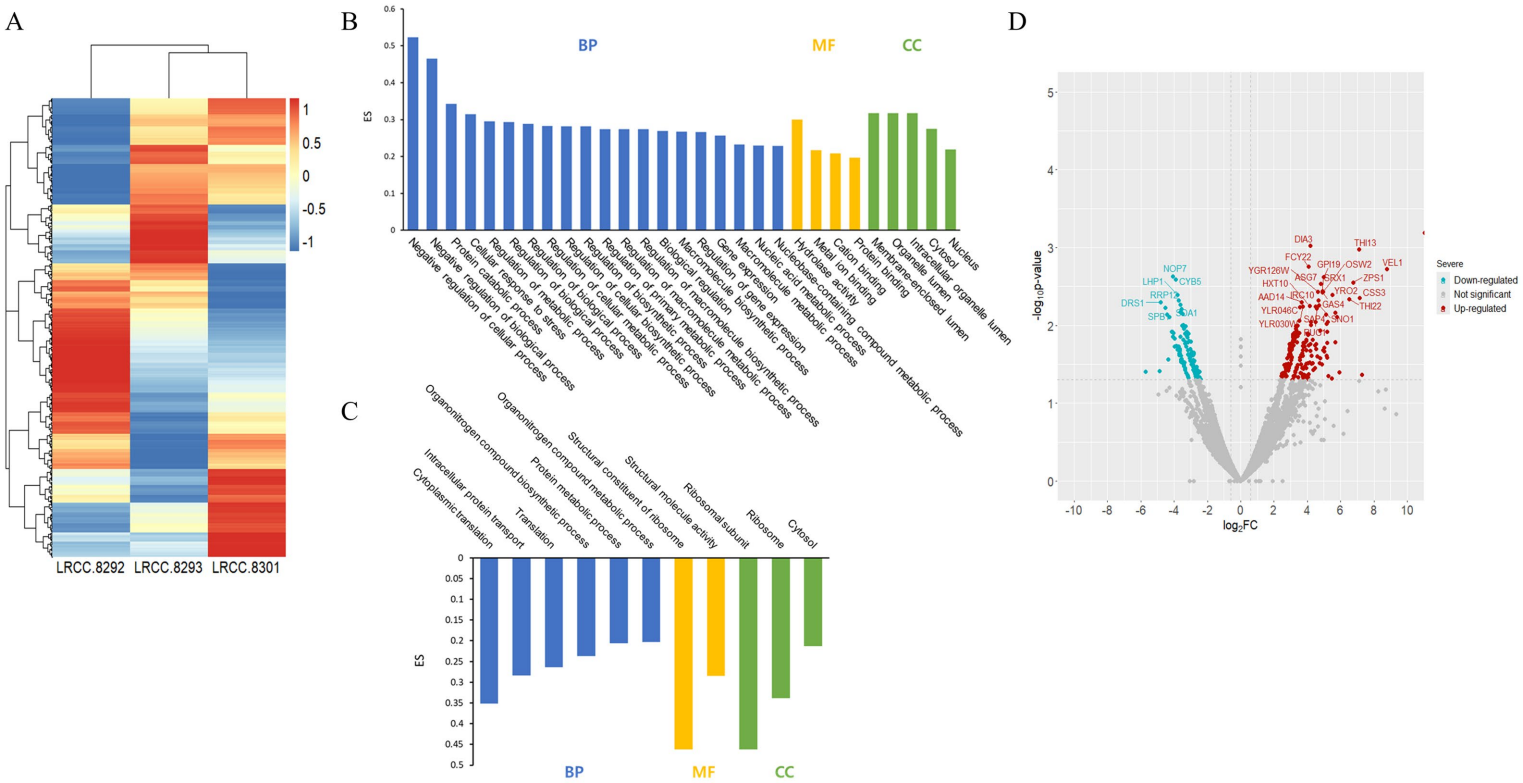
Global transcriptomic differences during alcoholic fermentation. **(A)** Heatmap of hierarchical clustering of differentially expressed genes (DEGs) across the three yeast strains. **(B)** Gene ontology (GO) enrichment analysis of upregulated and **(C)** downregulated genes in strain SC8292, categorized into biological process (BP), molecular function (MF), and cellular component (CC). The enrichment analysis highlights functional differences among strains associated with metabolic processes and cellular regulation. **(D)** Volcano plot of the distribution of DEGs identified by RNA-sequencing (RNA-seq) analysis in strain SC8292, with significantly upregulated or downregulated genes highlighted, demonstrating substantial transcriptional variation among strains.

**Figure 3:**
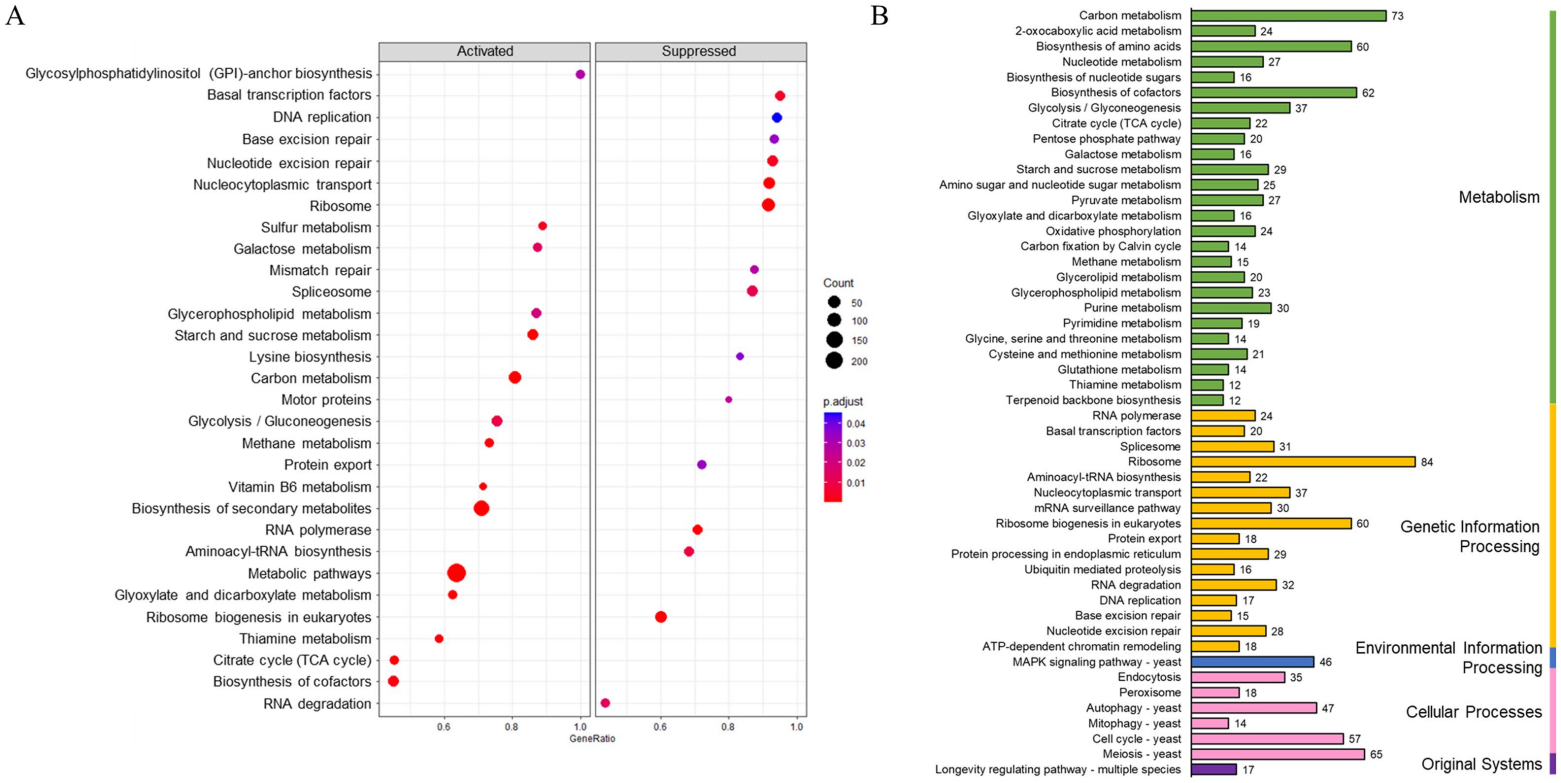
Functional enrichment analysis of DEGs in strain SC8292. **(A)** Bubble plot of the GO enrichment analysis of DEGs across the three yeast strains. The size of each bubble indicates the number of genes associated with each GO term; meanwhile, the color scale represents statistical significance. **(B)** Kyoto Encyclopedia of Genes and Genomes (KEGG) pathway enrichment analysis of the metabolic pathways significantly associated with the DEGs.

The overall transcriptional differences among the three strains were visualized using a heatmap of DEGs (Figure 2A), which clearly showed distinct clustering patterns and expression profiles for each strain. Functional grouping of DEGs based on the GO and KEGG databases was then performed to elucidate potential functional differences among strains. GO analysis of DEGs in SC8292 revealed significant alterations in the cellular component (CC), biological process (BP), and molecular function (MF) of both up- and downregulated genes (Figures 2B,C). In addition, the volcano plot of DEGs in SC8292 further illustrated the distribution of significantly upregulated and downregulated genes, indicating substantial transcriptional changes in this strain during fermentation (Figure 2D). KEGG pathway enrichment analysis of DEGs in SC8292 (Figure 3A) showed significant enrichment in various metabolic and cellular pathways. Upregulated genes were primarily associated with carbon metabolism, glycolysis/gluconeogenesis, general metabolic processes, and secondary metabolite biosynthesis, suggesting enhanced metabolic activity. In contrast, downregulated genes were predominantly involved in DNA replication, base excision repair, and ribosome biogenesis, indicating suppression of DNA maintenance and repair-related processes. A separate KEGG enrichment analysis of DEGs in SC8292 (Figure 3B) further revealed enrichment in pathways including carbon metabolism, meiosis, biosynthesis of cofactors, and biosynthesis of amino acids. Collectively, these transcriptomic differences suggest that strain-specific metabolic and regulatory activities may underlie the distinct VOC production patterns observed during fermentation. Corresponding analyses for SC8293 and SC8301 are presented in Supplementary Figures S1–S4.

### 3.4 Integration of multi-omics data

**Figure 4:**
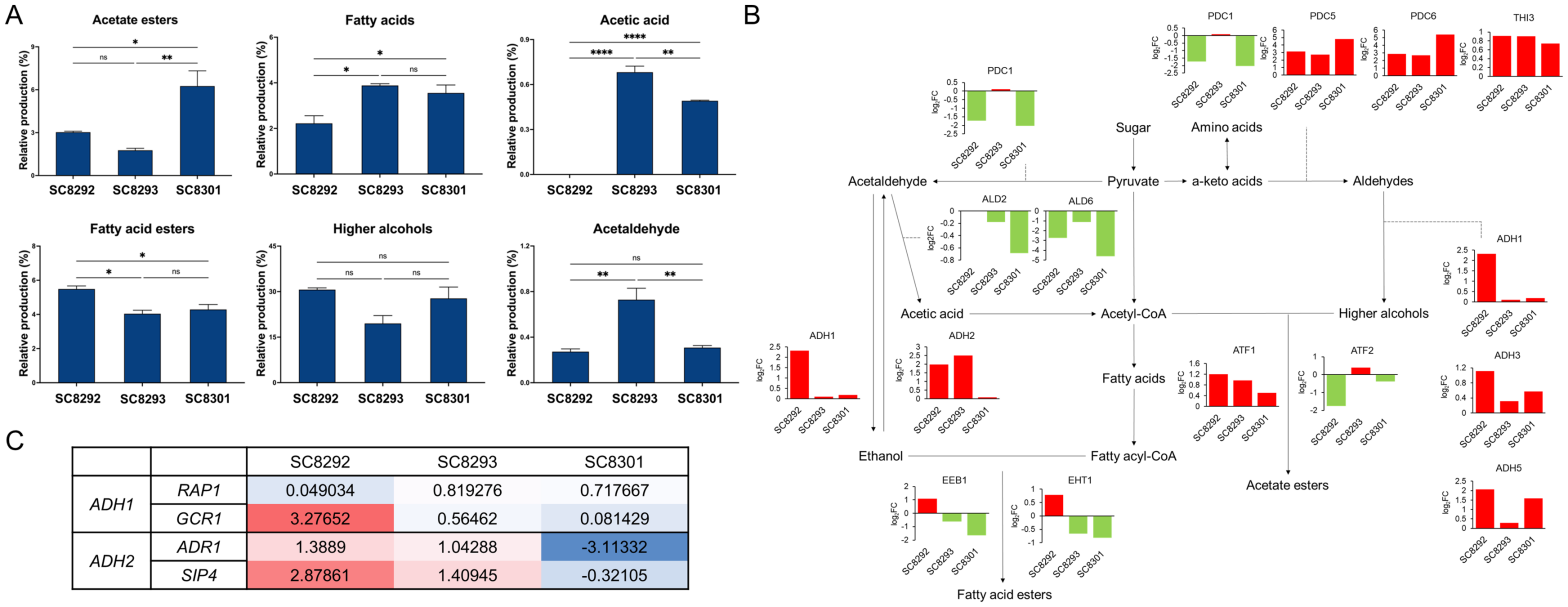
Integrated analysis of volatile compound production and transcriptional regulation of aroma-related genes. **(A)** GC–MS-based quantification of major VOCs produced during fermentation by three *S. cerevisiae* strains. The analyzed compounds include higher alcohols (e.g., isoamyl alcohol, isobutyl alcohol, phenethyl alcohol), acetate esters (e.g., isoamyl acetate, phenethyl acetate), fatty acid ethyl esters (e.g., ethyl octanoate, ethyl hexanoate), and undesirable compounds such as fatty acids and acetaldehyde. **(B)** RNA-seq-derived expression profiles (log₂ fold change) of genes involved in key metabolic pathways associated with volatile compound biosynthesis. Genes associated with the Ehrlich pathway and ester synthesis, including *ADH1*, *ADH3*, *ADH5*, *EEB1*, and *EHT1*, exhibited differential expression patterns among strains. **(C)** Expression levels of transcription factors regulating *ADH1* and *ADH2* transcription. Increased expression of *GCR1*, a transcriptional activator of *ADH1*, was observed in strain SC8292, whereas reduced expression of *ADR1* and *SIP4*, which regulate *ADH2* transcription, was detected in strain SC8301. These regulatory differences likely contribute to strain-specific variation in alcohol metabolism and VOCs production.

To identify potential molecular mechanisms underlying strain-specific aroma profiles, GC–MS and RNA-seq datasets were integratively analyzed by focusing on genes and metabolites associated with major VOC biosynthetic pathways. Among the VOCs analyzed by GC–MS, we focused on key aromatic compounds, including higher alcohols (isoamyl alcohol, isobutyl alcohol, phenethyl alcohol), acetate esters (isoamyl acetate, isobutyl acetate, phenethyl acetate), fatty acid esters (ethyl octanoate, ethyl hexanoate, ethyl decanoate, ethyl butyrate, ethyl 9-decenoate), and ethyl acetate, as well as compounds known to impart undesirable odors, such as fatty acids (decanoic acid, hexanoic acid, nonanoic acid, octanoic acid), acetic acid, and acetaldehyde (Figure 4A) (Heitmann et al., 2018). The association of these compounds with transcriptomic profiles obtained from RNA-seq was subsequently analyzed (Figure 4B).

Higher alcohols are formed via the Ehrlich pathway from amino acids, which involves three key enzymes: transaminase, decarboxylase, and alcohol dehydrogenase (Hazelwood et al., 2008). GC–MS analysis revealed that SC8292 produced the highest amount of higher alcohols. Consistently, RNA-seq results indicated that SC8292 exhibited elevated expression of alcohol dehydrogenase genes (*ADH1, ADH3*, and *ADH5*), which are involved in the reduction of aldehydes into higher alcohols during the Ehrlich pathway (Hazelwood et al., 2008). These findings suggest that enhanced expression of alcohol dehydrogenase-related genes may be associated with increased higher alcohol production in SC8292.

In the case of fatty acid esters, these compounds are synthesized from ethanol and acyl-CoA through the action of *EEB1* and *EHT1* genes (Rigou et al., 2021). Transcriptomic analysis showed that both *EEB1* and *EHT1* were highly expressed in SC8292, which corresponded with the highest production of fatty acid esters in this strain. This result can be further linked to ethanol biosynthesis, as ethanol is derived from acetaldehyde via *ADH1* gene activity (Boubekeur et al., 2001). Based on RNA-seq analysis, the elevated expression of *ADH1* in SC8292 was consistent with increased ethanol production, thereby providing more substrate for fatty acid ester formation. These integrated transcriptomic and metabolomic patterns suggest that coordinated regulation of ethanol metabolism and ester biosynthesis pathways may contribute to fatty acid ester accumulation in SC8292.

Acetaldehyde can be produced either from ethanol via *ADH2* gene or from pyruvate via pyruvate decarboxylase (*PDC1*) (Dzialo et al., 2017). RNA-seq results indicated that both *ADH2* and *PDC1* showed the highest expression in SC8293, consistent with GC–MS data showing the greatest acetaldehyde production in this strain. Thus, differences in the expression levels of *ADH1* and *ADH2* appear to be associated with differences in the production of acetaldehyde, ethanol, and fatty acid esters. These results suggest that strain-specific regulation of acetaldehyde metabolism may influence the accumulation of undesirable VOCs during fermentation.

To further investigate the differential expression of *ADH1* and *ADH2* among strains, transcription factors known to regulate these genes were examined. The *ADH1* promoter contains an upstream activating sequence (UAS) with a *RAP1* binding site. *RAP1* facilitates promoter accessibility, enabling *GCR1* to bind to this region and strongly activate *ADH1* transcription (Shore, 1994). In contrast, the promoter of *ADH2* contains an *ADR1* binding site (Walther and Schuller, 2001), where *ADR1* forms a complex with cofactors such as *SIP4* to activate transcription (Soontorngun et al., 2012). RNA-seq analysis revealed that *GCR1* expression was markedly elevated in SC8292, consistent with high *ADH1* expression, whereas both *ADR1* and *SIP4* expression were reduced in SC8301, which showed the lowest *ADH2* expression (Figure 4C). These findings suggest that *ADH1* and *ADH2* expression is modulated by the expression levels of the associated transcription factors.

**Figure 5:**
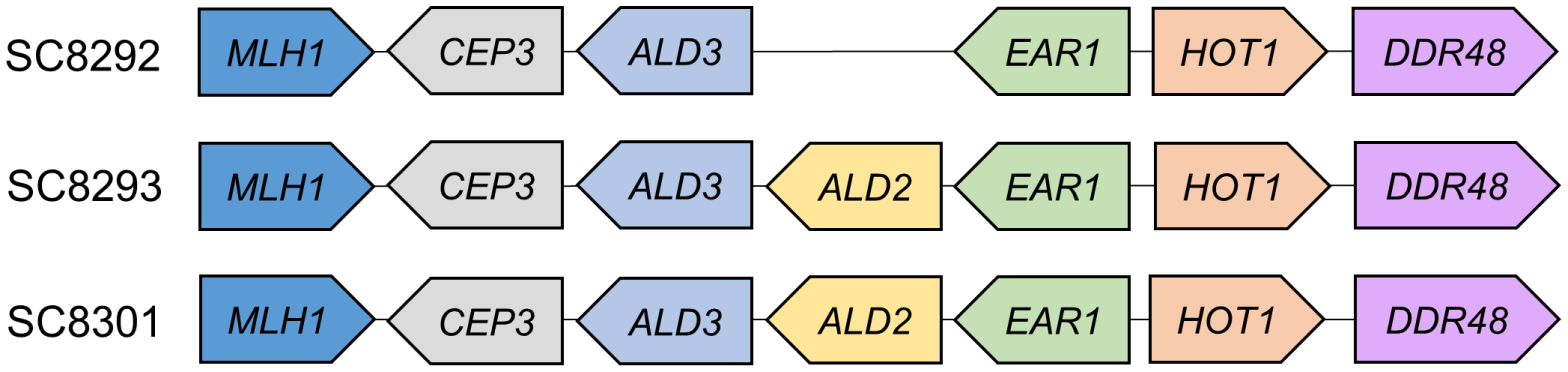
Comparative gene synteny analysis of the *ALD2* genomic region in three *S. cerevisiae* strains. Gene synteny analysis of the genomic region surrounding *ALD2*, which encodes an aldehyde dehydrogenase that converts acetaldehyde to acetate during fermentation. Conserved neighboring genes are present in strains SC8293 and SC8301, whereas the *ALD2* gene and associated homologous sequences are absent in strain SC8292. This structural genomic variation explains the absence of acetic acid production observed in strain SC8292 and highlights the critical role of *ALD2* in acetate biosynthesis during yeast fermentation.

Based on GC–MS data, acetic acid production was absent in SC8292 but present in the other strains. Acetic acid is typically produced from acetaldehyde through the oxidation activity of aldehyde dehydrogenase, encoded by *ALD2*, which links acetaldehyde metabolism to acetate-related VOC biosynthesis (Dzialo et al., 2017). Transcriptomic data indicated that *ALD2* expression was diminished but detectable in SC8293 and SC8301, whereas *ALD2* was not detected in the SC8292 genome (Table 4). Subsequent gene synteny analysis demonstrated that the *ALD2* gene, as well as any partial homologous sequences, were missing from the SC8292 genome (Figure 5). These findings are consistent with the absence of acetic acid production in SC8292 and suggest an important role of *ALD2* in acetic acid biosynthesis.

**Table 4:** *ALD2* gene in *S. cerevisiae* strains SC8292, SC8293, and SC8301.

Strain	*ALD2* gene ID	Chromosome	Positions	Length (bp)
SC8292	–	–	–	–
SC8293	LY8293002910	Chr13	573,319–574,839	1,521
SC8301	LY8301002897	Chr13	573,385–574,905	1,521

Acetate esters are synthesized from acetyl-CoA and higher alcohols by the action of alcohol acetyltransferases encoded by *ATF1* and *ATF2*. Previous work has shown that acetate ester production is more strongly influenced by enzyme activity and substrate availability than by *ATF1* and *ATF2* transcript levels (Verstrepen et al., 2003). In this study, acetate ester concentrations were highest in SC8301, followed by SC8292 and SC8293. No consistent correlation was observed between the expression of *ATF1* and *ATF2* and the levels of acetate esters. Instead, SC8301 exhibited the highest production of higher alcohols, thereby providing abundant substrates for ester formation, which likely contributed to the associated enhanced accumulation of acetate esters. Although SC8292 also produced substantial amounts of higher alcohols, SC8292 did not generate acetic acid—the precursor of acetyl-CoA—due to the absence of *ALD2*, as discussed above. Thus, the acetate ester production in SC8292 was likely lower than in SC8301. Collectively, these data support the conclusion that acetate ester biosynthesis is primarily modulated by substrate availability and enzyme functionality rather than by gene expression levels.

## 4 Discussion

The present study offers an integrative analysis of aroma formation in *S. cerevisiae* by linking genomic variation, transcriptional regulation, and volatile metabolite production. The results demonstrate that strain-specific differences in VOC profiles are strongly associated with variation in gene expression and genomic composition. Such strain-dependent metabolic diversity has been widely reported in fermentation yeasts and is recognized as a major driver of the sensory characteristics of fermented beverages (Styger et al., 2011; Pires et al., 2014).

One of the most notable findings of this study was the increased production of higher alcohols in SC8292. Higher alcohols are generated via the Ehrlich pathway, in which amino acids are converted to fusel alcohols through sequential transamination, decarboxylation, and reduction reactions. The elevated expression of *ADH1*, *ADH3*, and *ADH5* in this strain suggests an enhanced capacity to reduce aldehyde intermediates to the corresponding alcohols. Alcohol dehydrogenases play a central role in maintaining redox balance during fermentation and directly influence the accumulation of fusel alcohols (Dickinson et al., 2003). Therefore, increased activity of these enzymes may increase metabolic flux through the Ehrlich pathway, resulting in higher concentrations of aroma-active alcohols.

In addition to higher alcohol production, SC8292 exhibited increased levels of fatty acid ethyl esters. The upregulation of *EEB1* and *EHT1* suggests that these enzymes contribute significantly to ester biosynthesis in this strain. These acyltransferases catalyze the condensation of ethanol with medium-chain acyl-CoA molecules derived from fatty acid metabolism (Saerens et al., 2006). Previous studies have demonstrated that the expression levels of ester-forming enzymes strongly influence the accumulation of fruity VOCs during fermentation (Walther and Schuller, 2001). Therefore, the increased expression of these genes in SC8292 likely contributes to the enhanced ester production observed in this strain.

Another important observation was the accumulation of acetaldehyde and organic acids in SC8293. Transcriptomic analysis revealed increased expression of *ADH2* and *PDC1*, which encode enzymes involved in the conversion of pyruvate to acetaldehyde and the subsequent reduction of acetaldehyde to ethanol. Pyruvate decarboxylase (Pdc1p) catalyzes the decarboxylation of pyruvate to acetaldehyde, a key step in alcoholic fermentation. Elevated expression of this enzyme may increase intracellular acetaldehyde concentrations, which can subsequently be oxidized to acetate through aldehyde dehydrogenase activity (Saint-Prix et al., 2004). Increased acetaldehyde levels are known to contribute to undesirable sensory characteristics, such as pungent or green apple-like aromas, in fermented beverages.

Gene synteny analysis revealed that the *ALD2* gene is absent in SC8292. *ALD2* encodes an aldehyde dehydrogenase that converts acetaldehyde to acetate. Reduced activity or loss of ALD enzymes has previously been associated with decreased acetic acid formation during fermentation (Saint-Prix et al., 2004). Since excessive acetic acid production is a major cause of sour off-flavors in fermented beverages, the absence of *ALD2* may represent a beneficial genetic trait in strains developed to produce improved aroma profiles during fermentation.

In addition to metabolic enzymes, transcriptional regulators appear to play an important role in coordinating strain-specific VOC biosynthesis during fermentation. The elevated expression of *GCR1* observed in SC8292 may enhance glycolytic flux, as Gcr1p functions as a transcriptional regulator of glycolytic gene expression. Increased glycolytic activity would raise pyruvate and acetyl-CoA levels, providing key precursors for ethanol production and ester biosynthesis. Conversely, the reduced expression of regulatory genes such as *ADR1* and *SIP4* in SC8301 suggests altered regulation of ethanol metabolism and gluconeogenic pathways. Such regulatory variation may influence carbon flux distribution and ultimately affect the balance among ethanol production, higher alcohol synthesis, organic acid accumulation, and overall VOC composition during fermentation. These findings suggest that strain-specific transcriptional regulation may contribute to the coordinated metabolic differences observed among the *S. cerevisiae* strains.

The integration of genomic, transcriptomic, and metabolomic datasets highlights the complex regulatory architecture underlying aroma formation during yeast fermentation. While biochemical pathways such as the Ehrlich pathway and fatty acid metabolism provide the metabolic framework for VOC production, the final aroma profile emerges from the interplay among enzyme expression levels, metabolic flux distributions, and regulatory network architecture. Therefore, systems-level approaches that integrate multiple omics datasets are essential for understanding the genetic basis of fermentation phenotypes (Bordet et al., 2020).

From an industrial perspective, identifying genes associated with aroma production has important implications for strain selection and metabolic engineering. The candidate genes identified in this study, including *ADH1*, *ADH2*, *EEB1*, *EHT1*, and *ALD2*, may serve as molecular markers for selecting yeast strains that produce desirable flavor profiles. In addition, targeted genetic modifications aimed at increasing ester production while reducing aldehyde or volatile acid accumulation could improve fermentation quality in industrial processes. Previous studies on commercial brewing yeasts have demonstrated substantial strain-dependent differences in VOC production, particularly in higher alcohol and ester biosynthesis. Ale-type *S. cerevisiae* strains are generally associated with elevated ester and higher alcohol production compared with lager yeasts, reflecting differences in fermentation metabolism and transcriptional regulation (Pires et al., 2014; Paszkot et al., 2023). Consistent with these observations, SC8292 exhibited increased expression of alcohol dehydrogenase-related genes together with elevated higher alcohol and ester-associated VOC profiles, suggesting that this strain possesses aroma-associated metabolic characteristics similar to highly fermentative brewing yeasts.

Previous QTL and yeast aroma genetics studies have identified multiple genes associated with strain-specific VOC production during alcoholic fermentation, including alcohol acetyltransferases (*ATFs*) and ester biosynthesis-related genes such as *EEB1* and *EHT1* (Eder et al., 2018). Consistent with these previous findings, the present transcriptomic and metabolomic analyses also indicated elevated expression of ester-associated genes together with increased fatty acid ester production in SC8292. Previous studies on acetate metabolism during alcoholic fermentation have primarily emphasized the role of *ALD6* as a major contributor to acetate biosynthesis (Ho et al., 2021). However, the strain-specific absence of *ALD2* together with the lack of detectable acetic acid production in SC8292 represents a potentially distinct observation that may reflect altered aldehyde metabolism in this strain. These findings suggest that *ALD2*-associated pathways may also contribute to strain-specific acetate-related VOC formation, although further mechanistic investigation will be necessary to clarify its precise role during fermentation.

Integrated transcriptomic and metabolomic analyses in wine and brewing yeasts have previously been used together with correlation- or network-based approaches to investigate aroma-associated metabolic pathways (Rossouw et al., 2008; Mendes et al., 2017). In comparison, this study focused on exploratory comparative multi-omics profiling to identify potential associations between strain-specific transcriptional patterns and VOC production under standardized fermentation conditions. Variations in fermentation systems, VOC extraction methodologies, and analytical strategies among studies may also contribute to differences in the reported metabolic and transcriptomic relationships. Future studies should further investigate the functional roles of these genes using targeted genetic approaches such as gene deletion, overexpression, and clustered regularly interspaced short palindromic repeats (CRISPR)-mediated genome editing. Based on the present observations, targeted manipulation of candidate genes may provide further insight into strain-specific VOC biosynthesis pathways. For example, CRISPR-mediated disruption of *ALD2* may result in reduced or absent acetic acid production, similar to the phenotype observed in SC8292, whereas altered expression of ester biosynthesis genes such as *EEB1* and *EHT1* may affect fatty acid ester accumulation during fermentation. Such functional studies would help clarify the mechanistic relationships between transcriptomic regulation and aroma-associated metabolite formation. Although the metabolomic analyses were performed in biological triplicate, the transcriptomic analysis was conducted using single biological samples for each condition. Therefore, the RNA-seq results should be interpreted as exploratory findings that identify potential molecular associations requiring further validation through independent biological replicates and functional studies. Additionally, environmental factors, including fermentation temperature, oxygen availability, and nutrient composition, are known to influence metabolic flux and the formation of VOCs (Swiegers and Pretorius, 2005). Investigating how these environmental variables interact with genetic background will be essential for fully elucidating the regulation of aroma metabolism in yeast fermentation systems. And because the present study was designed as an exploratory multi-omics investigation, the observed associations between gene expression and VOC production should be interpreted as hypothesis-generating rather than causal relationships. Future studies incorporating biological replicates, formal statistical modeling approaches such as regression or network analysis, and functional validation experiments will be necessary to establish robust mechanistic relationships.

## Data Availability

The datasets presented in this study can be found in online repositories. The names of the repository/repositories and accession number(s) can be found at: https://www.ncbi.nlm.nih.gov/genbank/, PRJNA1439535.
