# Supplementary material for: Integrative multi-omics reveals genetic and transcriptomic determinants of aroma formation during alcoholic fermentation in Saccharomyces cerevisiae
Source: Front Microbiol. 2026 May 29;17:1866172. doi: 10.3389/fmicb.2026.1866172 (PMC13260619; doi:10.3389/fmicb.2026.1866172)
Supplement: Supplementary file 1 [file Data_Sheet_1.pdf]

frontiers

Supplementary materials

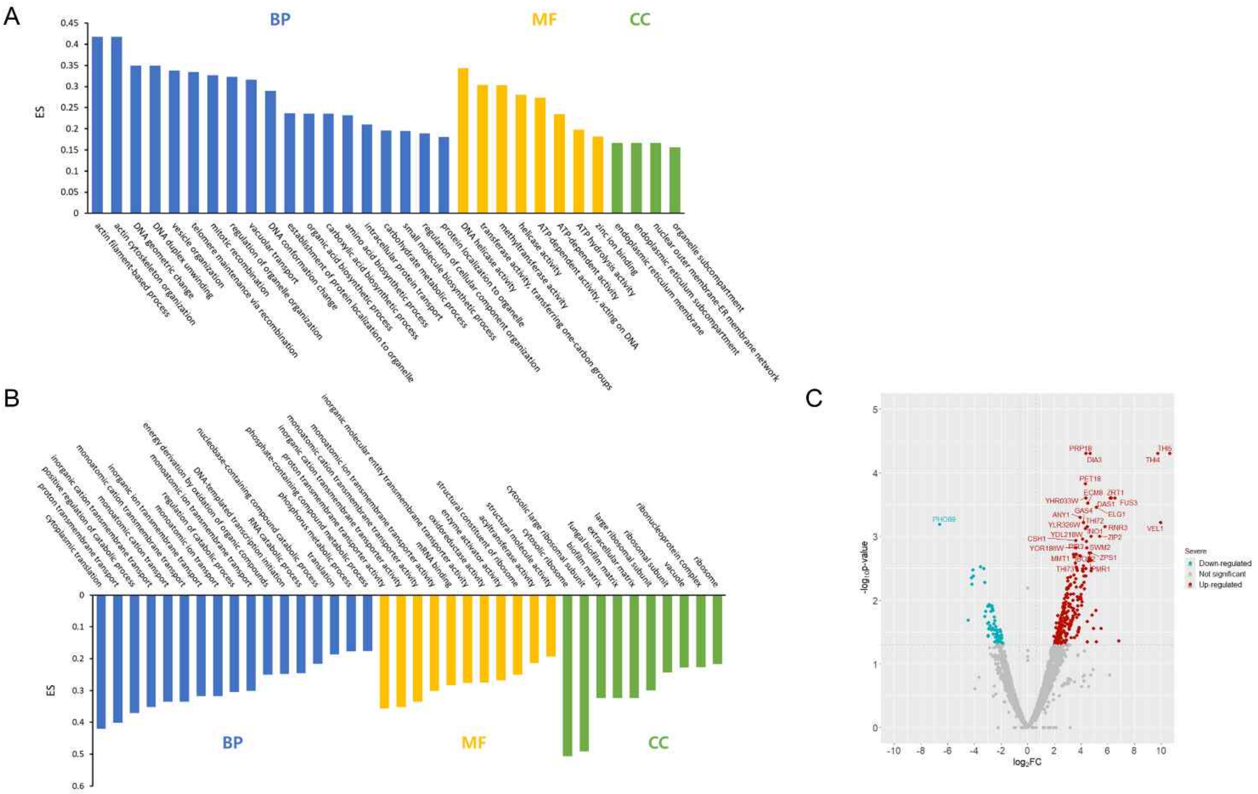

Supplementary Figure 1. (A) GO enrichment of up-regulated genes and (B) down-regulated genes, and (C) volcano plot of DEGs for SC8293

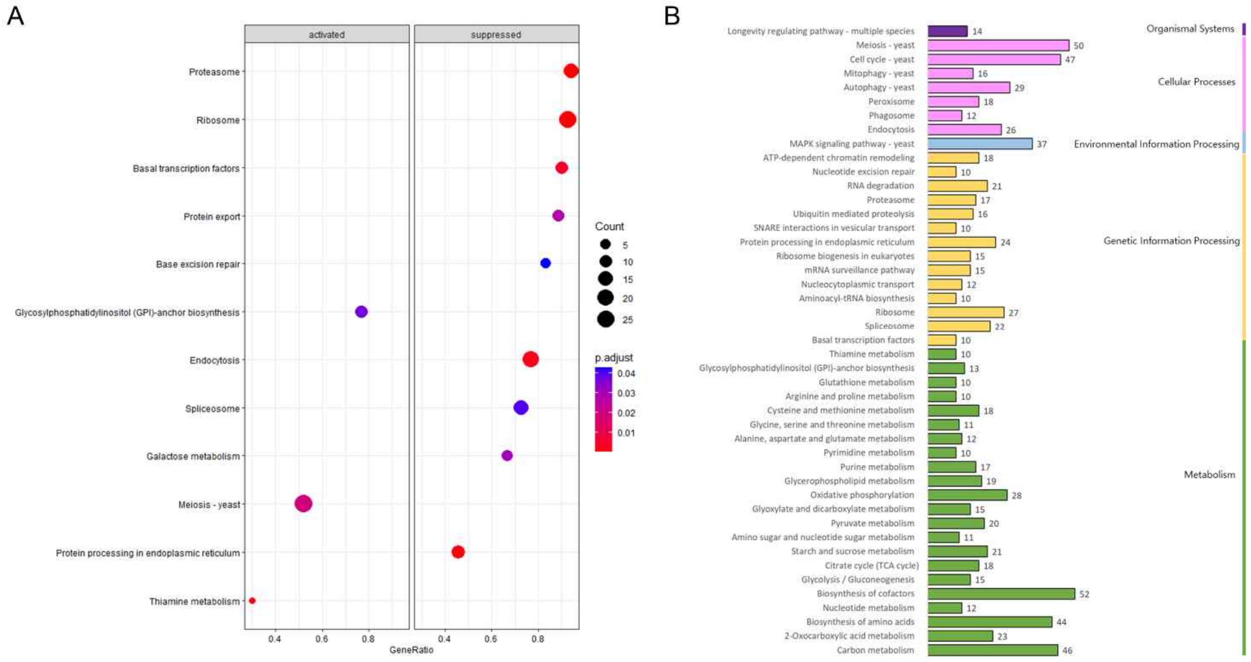

Supplementary Figure 2. (A) Bubble plot of GO annotation and (B) KEGG enrichment pathway analysis of SC8293.

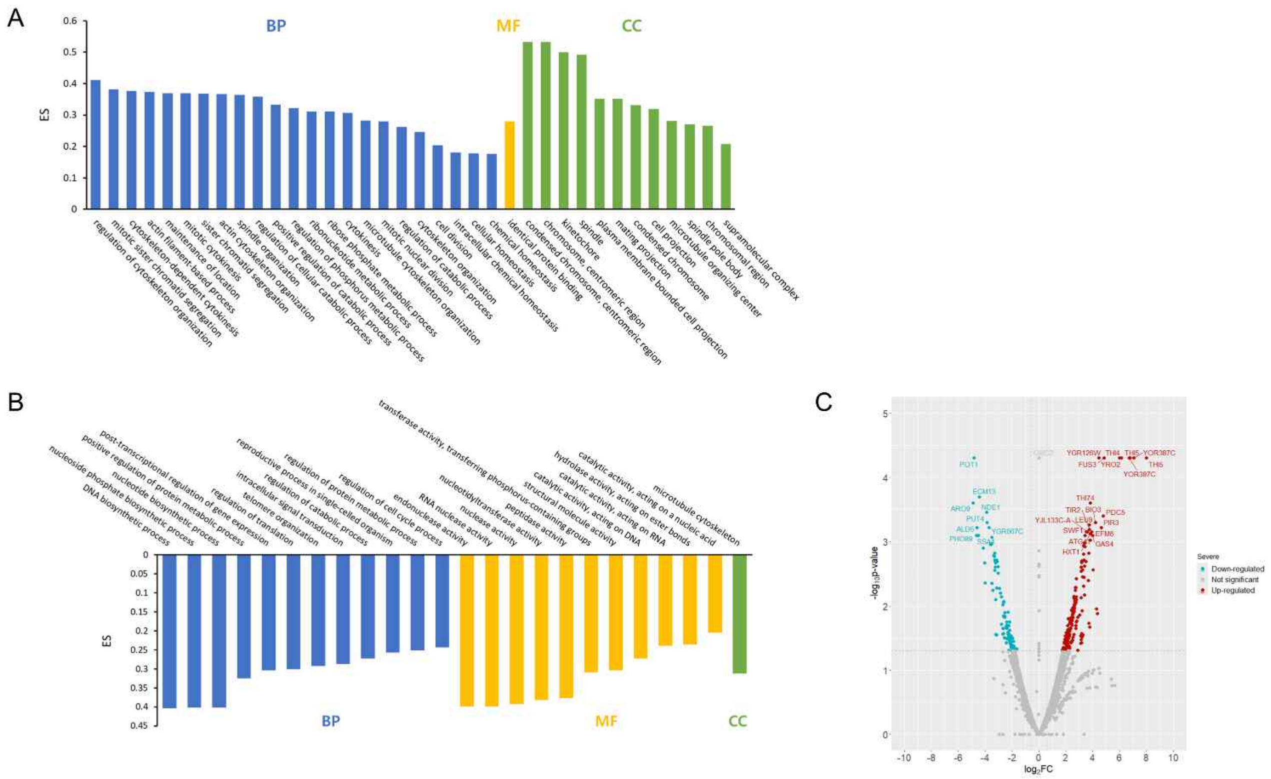

Supplementary Figure 3. (A) GO enrichment of up-regulated genes and (B) down-regulated genes, and (C) volcano plot of DEGs for SC8301.

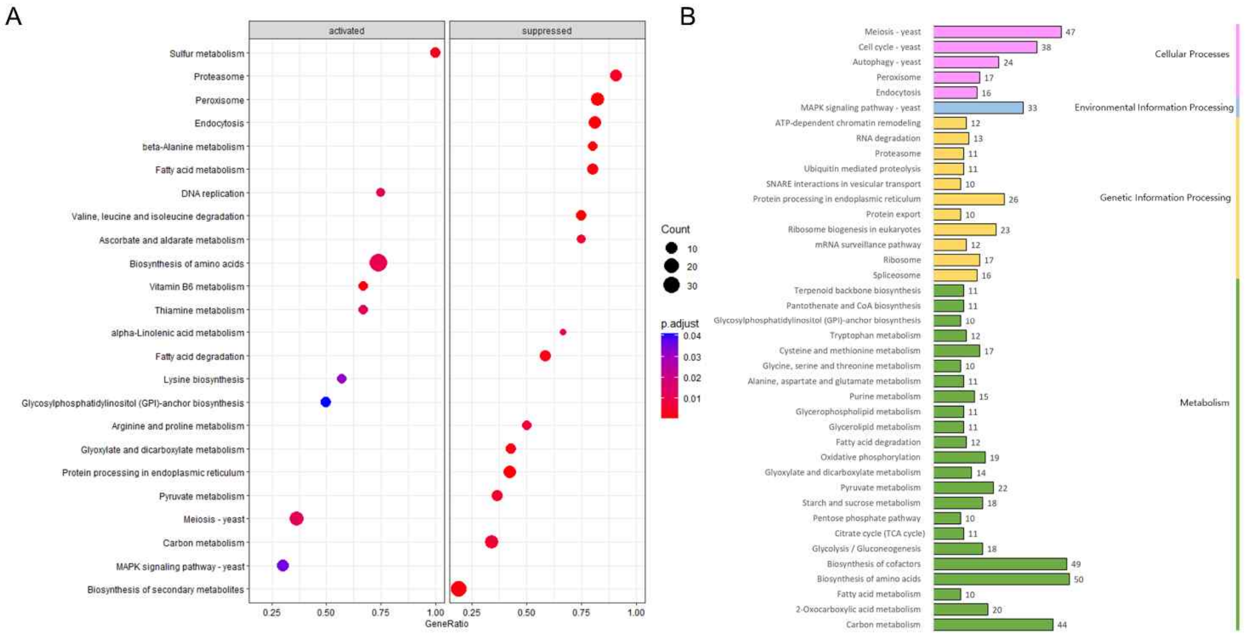

Supplementary Figure 4. (A) Bubble plot of GO annotation and (B) KEGG enrichment pathway analysis of SC8301.
